# A Pilot Study of AI-Controlled Transcutaneous Peripheral Nerve Stimulation for Essential Tremor

**DOI:** 10.5334/tohm.991

**Published:** 2025-03-21

**Authors:** Richard Dewey, Stuart Isaacson, Richard Dewey, Sagari Betté, Kelly E. Lyons, Zhi Yang, Anh Tuan Nguyen, Qi Zhao, Zhen Zhang, Rajesh Pahwa

**Affiliations:** 1 Parkinson’s Disease and Movement Disorders Center of Boca Raton, Boca Raton, FL, US; 2 University of Kansas Medical Center, Department of Neurology, Kansas City, KS, US; 3 Fasikl Inc., US; 4 University of Minnesota, US

**Keywords:** transcutaneous peripheral nerve stimulation, artificial intelligence, TETRAS scale, neuromodulation, central tremor network

## Abstract

**Background:**

Essential tremor (ET) can impact daily activities and quality of life. Transcutaneous peripheral nerve stimulation (TPNS) modulates the central tremor network and can reduce tremor. We report a pilot study with a novel TPNS device.

**Methods:**

In this prospective, open-label study, ET patients underwent tremor evaluation and device fitting in the clinic, then used the system at home during waking hours for 7 to 10 days. Efficacy outcomes were the change from baseline to follow-up in The Essential Tremor Rating Assessment Scale (TETRAS) Performance Subscale (PS) for upper limbs, the TETRAS Activities of Daily Living (ADL) Subscale, the modified ADL (mADL) Score, and the Patient and Clinician Global Impression of Improvement questionnaires (PGI-I, CGI-I). Safety was also assessed.

**Results:**

In the 17 patients with evaluable data, the dominant-hand PS improved from 14.1 at baseline to 11.4 at follow-up (p = 0.0002); the ADL and mADL improved from 29.9 to 20.7 and 34.8 to 24.8, respectively (both p < 0.001). Improvement was reported for 82% of patients on both the PGI-I and CGI-I. A skin reaction in one patient with adhesive allergy was the only adverse event.

**Discussion:**

AI-controlled TPNS shows promise as a safe and effective treatment option for ET patients.

**Highlights:**

In an uncontrolled pilot study, an AI-controlled transcutaneous peripheral nerve stimulation device was worn continuously during waking hours for 7 to 10 days by patients with essential tremor. Tremor statistically significantly decreased as measured by TETRAS subscales and Global Impression of Improvement questionnaires, and side effects were negligible.

## Introduction

Essential tremor (ET), one of the most common movement disorders, is characterized by involuntary, rhythmic, oscillatory movements [1]. Tremor in the hands can interfere with many daily activities, such as drinking, eating, and writing, which impairs quality of life and may lead to disability and social isolation. The postural and kinetic tremor seen in ET predominately affects the upper limbs but may also occur in the head, lower limbs, voice, tongue, face, and trunk [2]. Approximately 0.9% of people worldwide have ET [3]; in the United States, approximately 7 million are affected [4].

The mechanism underlying tremor in ET is unknown but is thought to relate to oscillatory activity within a central tremor network involving the ventral intermediate nucleus of the thalamus (VIM) [5,6]. Propranolol and primidone are the medications used most frequently and successfully to treat ET, and propranolol is the only medication approved by the US Food and Drug Administration (FDA) for ET. However, approximately 30–50% of patients will not respond to either propranolol or primidone, and many experience adverse effects [7,8]. Moreover, even in first-line drug responders, the reduction in postural tremor amplitude is only about 50–60% [9]. Patient responses to second-line medications for ET, including topiramate, benzodiazepines, gabapentin, zonisamide, and pregabalin, are variable [4]. Surgical options include deep brain stimulation (DBS) of the VIM and magnetic resonance–guided focused ultrasound VIM thalamotomy; although often effective, these options can carry the significant safety risks and expenses associated with invasive procedures [10]. In addition, not all persons with ET may be candidates for a surgical procedure [11,12].

Both sensory and motor inputs contribute to the pathogenesis of ET [13,14], and numerous studies have examined the ability of peripheral stimulation of nerves or muscles to lessen the tremor [15]. One device, the Cala system, based on transcutaneous afferent patterned stimulation (TAPS), has 510(k) clearance by the FDA for the treatment of ET [16]. This system alternately stimulates the median and radial nerves in the wrist at a frequency tuned to the patient’s tremor to evoke activity within VIM and other structures within the central tremor network [10,17].

Here we report a pilot study of a novel transcutaneous peripheral nerve stimulation (TPNS) device. The TPNS system is different from TAPS devices in several ways: First, its operation is powered by cloud-based artificial intelligence (AI) that continuously adjusts stimulation based on patient activity and predictive analytics. Unlike the patterned cyclic stimulation used in TAPS devices, the TPNS device delivers stimulation with a personalized and context-dependent approach. Second, while traditional devices are typically used in one or two brief sessions per day to provide temporary tremor relief, AI in the TPNS system enables consistent symptom relief throughout the waking day, effectively addressing the challenges of neural adaptation associated with prolonged stimulation. Third, Felix is specifically designed for all-day use, ensuring that the stimulation does not interfere with users’ normal motor control or daily activities. Lastly, it stimulates all three peripheral nerves—radial, median, and ulnar—which contain both sensory and motor fibers, in contrast to traditional devices that focus solely on sensory stimulation.

## Methods

### Study design

This was a prospective, open-label, two-center pilot study designed to evaluate the safety and effectiveness of the TPNS system in ET patients (ClinicalTrials.gov number: NCT05842434). Patients underwent device fitting and tremor evaluation at baseline, then used the device at home during waking hours for 7 to 10 days, after which they returned to the clinic for follow-up. Physicians and patients jointly decided which hand to stimulate (dominant or nondominant) or whether to stimulate both hands by using 2 devices.

### Participants

Participants with ET who visited the University of Kansas Medical Center and the Parkinson’s Disease and Movement Disorders Center of Boca Raton were recruited for this study.

Key inclusion criteria were 1) at least 18 years of age; 2) willing to provide written, informed consent to participate in the study; 3) a clinical diagnosis of ET (a diagnosis in the medical record was acceptable); 4) for either upper limb, a tremor severity score of 2 or higher as measured by one of the TETRAS Performance Subscale (PS) upper limb items (items 4a, b, c: forward outstretched posture, lateral “wing beating” posture, finger-nose-finger; 6: Archimedes spiral drawing; 7: handwriting; and 8: dot approximation task) and a total score of 7 or higher across all TETRAS upper limb items; 5) stable dosage of any medication, if applicable, for 30 days prior to study entry; and 6) familiar with operating a touch-screen smartphone and connecting to Wi-Fi internet at home. Key exclusion criteria were 1) prior limb amputation or any known symptomatic peripheral neuropathy condition of the involved upper extremity; 2) any current drug or alcohol abuse; 3) current unstable epileptic condition with a seizure within 6 months of study entry; 4) pregnant or nursing subjects and those who planned pregnancy during the course of the study; 5) swollen, infected, or inflamed areas or skin eruptions, open wounds, or cancerous lesions of skin at the stimulation site; 6) known allergy to adhesives; 7) history of Alzheimer’s disease or dementia (Montreal Cognitive Assessment ≤19) [18]; 8) botulinum toxin injections for hand tremor within 4 months prior to study enrollment. Caffeine, alcohol, and marijuana use were permitted, but caffeine use was to remain stable over the course of the study; no alcohol was permitted on the day before each study visit; and use of alcohol or marijuana at levels considered by the physician to be abuse was reason for exclusion.

This study was approved by the Western-Copernicus Group Institutional Review Board. All participants provided written informed consent.

### Study device

**Figure 1:**
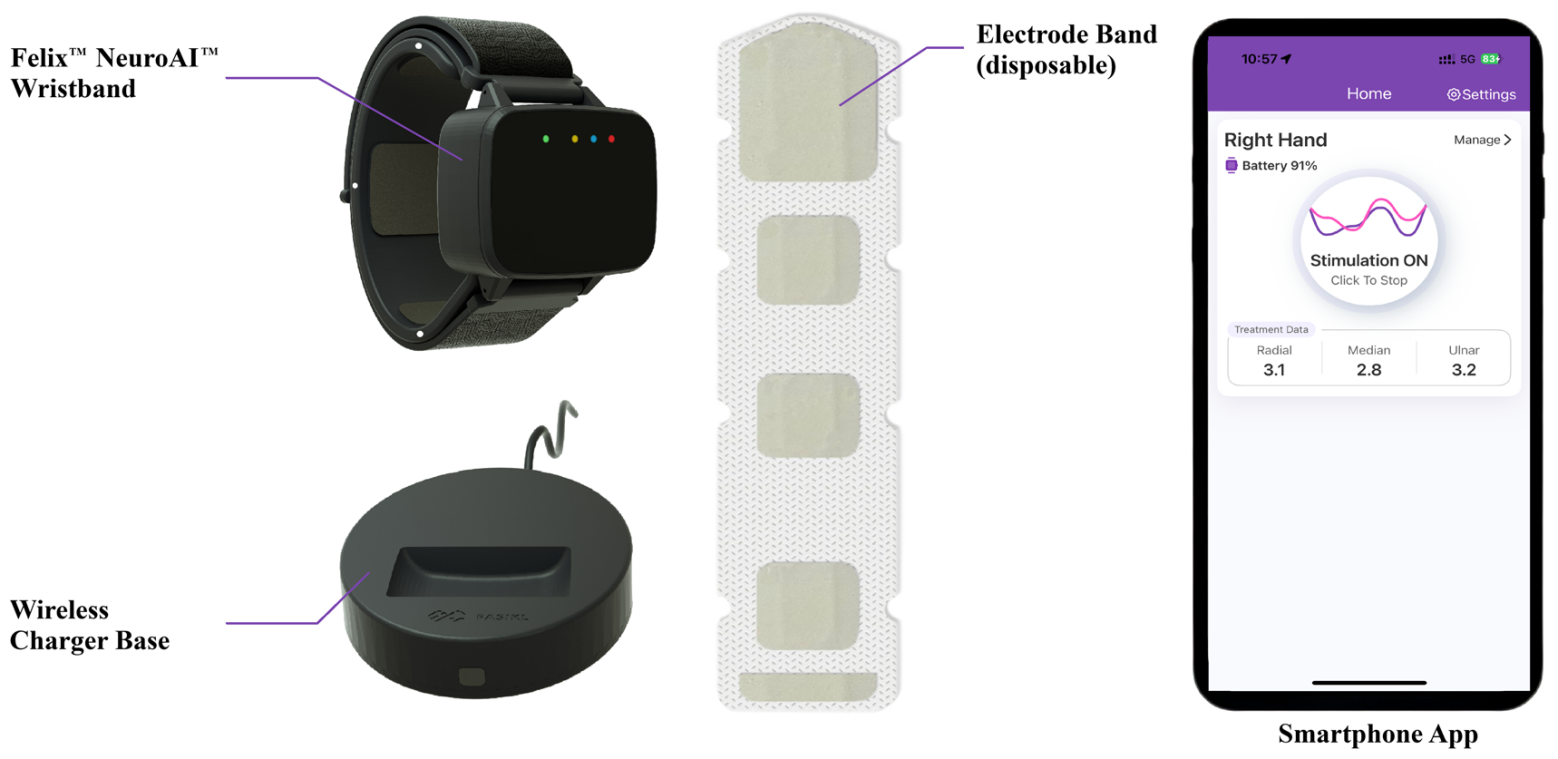
The components of the TPNS system. The TPNS system consists of a stimulator that contains all of the stimulator circuitry, sensors, Bluetooth communication, and battery. Three buttons on the side control power (on/off) and stimulation amplitude. The stimulator connects magnetically to a connector band, which itself fits inside a strap that is used to attach the device around the wrist. A disposable (single-use) electrode band is first wrapped around the wrist, followed by the stimulator/band/strap assembly. The connector band provides electrical connections between the stimulator and electrode band. Several different sizes of electrode and connector bands are available. The stimulator is controlled by a smartphone app via Bluetooth; it sends real-time inertial measurement unit data to the app and receives stimulation settings. When not in use, the stimulator can be charged with the wireless charger.

The TPNS system (Felix^™^ NeuroAI^™^ Wristband; Figure 1) is a neuromodulation device that stimulates the radial, median, and ulnar nerves [1]. The device records movement signals from the patient’s wrist through an inertial measurement unit with an accelerometer and gyroscope and uses a cloud-based AI algorithm to extract relevant parameters streamed from the device. The system continuously adjusts stimulation settings, such as pulse counts, stimulation sequence (between nerves), and stimulation intensity, in real time to optimize control of each individual’s tremor. It is designed to be worn by the patient on the wrist throughout the waking day.

### Assessments and outcomes

The Essential Tremor Rating Assessment Scale (TETRAS) [19] is an investigator-administered, pen and paper–based quantitative scale with Activities of Daily Living (ADL) and Performance (PS) Subscales. Each item of the subscales has a rating of 0 to 4, indicating increasing tremor severity. The outcomes of this study were the change from baseline to follow-up in the TETRAS PS Score for upper limb items (items 4a, b, c: forward outstretched posture, lateral “wing beating” posture, finger-nose-finger; 6: Archimedes spiral drawing; 7: handwriting; and 8: dot approximation task), all assessed bilaterally except for handwriting; the TETRAS ADL Subscale score; and the modified ADL (mADL) score, a sum of items 2 to 11 of the TETRAS ADL Subscale plus item 6 (bilateral) and item 7 (dominant hand) of the TETRAS Performance Subscale. The Patient and Clinician Global Impression of Improvement questionnaires (PGI-I and CGI-I, respectively) were also administered at follow-up. Device- and therapy-related adverse events were collected.

## Results

### Participants

Between June and November 2023, 25 ET patients were enrolled into the study. The mean duration of ET was 26.7 years, and 13 (52%) were women. Twenty (80%) had a family history of ET, and 22 (88%) were taking medication for ET. Of the patients enrolled, 18 (72%) wore the device on the dominant hand, 5 (20%) on the nondominant hand, and 2 (8%) got bilateral stimulation. Table 1 presents demographic information on all subjects as well as the cohort with evaluable data (n = 17).

**Table 1:** Participant demographics and clinical characteristics.


CHARACTERISTIC	ALL ENROLLED PARTICIPANTS (n = 25)	PARTICIPANTS WITH EVALUABLE DATA (n = 17)

Age (years)	71.0 ± 7.7 [53.0–87.0]	70.4 ± 8.1 [53.0–87.0]

Female	52 (13)	47 (8)

Race		

White	92 (23)	88 (15)

Black or African American	8 (2)	12 (2)

Ethnicity		

Hispanic or Latino	4 (1)	6 (1)

Non-Hispanic or Latino	96 (24)	94 (16)

BMI (kg/m^2^)	26.1 ± 4.2 [18.4 –37.7]	26.4 ± 3.5 [20.7–31.5)

Family history of ET	80 (20)	77 (13)

ET onset age (years)	44.3 ± 19.2 [10.0–78.0]	44.3 ± 19.2 (19.0–78.0)

Prior ET treatment	72 (18)	65 (11)

On ET medication	88 (22)	94 (16)

Response to Alcohol		

Yes	56 (14)	53 (9)

No	12 (3)	12 (2)

Unsure	32 (8)	35 (6)

Response to Caffeine		

Yes	40 (10)	41 (7)

No	20 (5)	18 (3)

Unsure	40 (10)	41 (7)

Tremor Severity		

Mild	32 (8)	29 (5)

Moderate	60 (15)	65 (11)

Severe	8 (2)	6 (1)

Stimulation		

Dominant Hand	72 (18)	88 (15)

Nondominant Hand	20 (5)	6 (1)

Both Hands	8 (2)	6 (1)


Qualitative data are presented as percentage (number of patients); quantitative data are presented as mean ± standard deviation [range].

**Figure 2:**
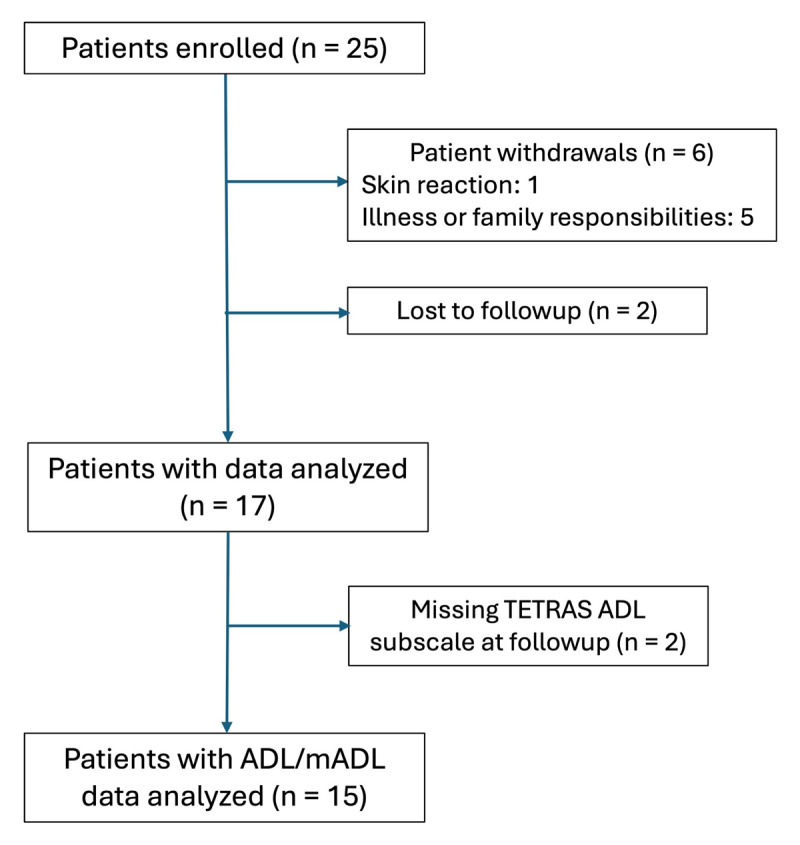
Flow diagram of patient progress through the study.

Six of the 25 patients (24%) withdrew from the study, one due to a skin reaction and the others due to participant illness (COVID-19) or family responsibilities. Two patients (8%) were lost to follow-up, and 2 patients were missing TETRAS ADL Subscale assessments at the follow-up visit (Figure 2).

### Outcomes

In the subset of patients who completed the follow-up visit (n = 17), the dominant hand PS improved from 14.1 at baseline to 11.4 at follow-up (p = 0.0002); the nondominant hand PS improved from 9.2 at baseline to 7.8 at follow-up (p = 0.006) (Table 2). The ADL and mADL also improved from 29.9 to 20.7 and 34.8 to 24.8, respectively (both p < 0.001). The majority of individual tasks assessed with the TETRAS Performance and ADL Scales also saw statistically significant improvements (Table 2). An analysis by stimulated and nonstimulated hand showed very similar results.

**Table 2:** Improvement on TETRAS subscales and individual items from baseline to day 7.


TETRAS SUBSCALE AND ITEM	BASELINE	FOLLOW-UP	CHANGE	p VALUE

TETRAS PS Score Dominant Hand (n = 17) ^a^	14.1 ± 2.2	11.4 ± 3.3	–2.7 ± 2.3	<0.001

TETRAS PS Score Nondominant Hand (n = 16) ^b^	9.2 ± 2.8	7.8 ± 2.7	–1.5 ± 1.8	0.006

TETRAS ADL Score (n = 15) ^c^	29.9 ± 4.2	20.7 ± 8.7	–9.3 ± 7.5	<0.001

TETRAS mADL Score (n = 15) ^d^	34.8 ± 4.8	24.8 ± 9.5	–10.0 ±7.4	<0.001

**PS Ratings**				

Dominant Hand				

Forward Outstretched Posture	2.1 ± 0.5	1.7 ± 0.5	–0.4 ± 0.6	0.0112

Lateral “Wing Beating” Posture	2.4 ± 0.6	1.9 ± 0.7	–0.5 ± 0.6	0.0030

Finger-Nose-Finger	2.0 ± 0.4	1.6 ± 0.6	–0.4 ± 0.5	0.0180

Archimedes Spiral Drawing	2.6 ± 0.5	2.3 ± 0.8	–0.4 ± 0.7	0.0479

Handwriting	2.6 ± 0.9	2.0 ± 1.0	–0.6 ± 0.6	0.0022

Dot Approximation	2.4 ± 0.5	1.9 ± 0.5	–0.5 ± 0.4	<0.001

Non-Dominant Hand				

Forward Outstretched Posture	1.4 ± 0.7	1.1 ± 0.7	–0.3 ± 0.7	0.1262

Lateral “Wing Beating” Posture	1.6 ± 0.8	1.3 ± 0.7	–0.2 ± 0.6	0.1347

Finger-Nose-Finger	1.8 ± 0.7	1.4 ± 0.7	–0.4 ± 0.8	0.0754

Archimedes Spiral Drawing	2.2 ± 0.9	2.1 ± 0.9	–0.2 ± 0.5	0.1881

Dot Approximation	2.2 ± 0.6	1.8 ± 0.4	–0.3 ± 0.4	0.0034

**ADL Ratings**				

Speaking	0.9 ± 1.0	0.8 ± 1.0	–0.1 ± 0.7	0.7192

Feeding with a spoon	3.1 ± 0.4	2.0 ± 0.9	–1.1 ± 0.9	<0.001

Drinking from a glass	2.9 ± 0.6	1.8 ± 1.1	–1.1 ± 1.1	0.0032

Hygiene	2.5 ± 0.7	1.5 ± 1.1	–0.9 ± 1.1	0.0077

Dressing	1.9 ± 0.8	1.3 ± 0.9	–0.6 ± 0.8	0.0140

Pouring	2.9 ± 0.8	2.1 ± 1.0	–0.8 ± 1.3	0.0342

Carrying food trays, plates or similar items	3.0 ± 0.7	2.3 ± 1.1	–0.7 ± 1.0	0.0156

Using keys	2.5 ± 1.0	1.4 ± 1.1	–1.1 ± 0.9	<0.001

Writing	2.7 ± 0.8	2.0 ± 1.0	–0.7 ± 1.0	0.0156

Working	2.5 ± 0.7	1.7 ± 0.9	–0.9 ± 0.9	0.0025

Overall disability with the most affected task	3.1 ± 0.3	2.2 ± 0.8	–0.9 ± 0.8	<0.001

Social Impact	2.0 ± 1.0	1.6 ± 1.1	–0.4 ± 0.8	0.0824


Data are presented as mean ± standard deviation, p values were obtained with paired sample t-tests. TETRAS PS Score for the dominant hand includes TETRAS PS items 4, 6, 7, 8. TETRAS PS Score for the non-dominant hand includes TETRAS PS items 4, 6, 8. Abbreviations: ADL, activities of daily living; mADL, modified activities of daily living; PS, Performance Scale; TETRAS, The Essential Tremor Rating Assessment Scale. ^a^Maximum score, 24. ^b^Maximum score, 20. ^c^Maximum score, 48. ^d^Maximum score, 52.

Improvement on PGI-I was reported by 82% of patients. Similarly, physicians reported on the CGI-I that 82% of patients showed improvement.

### Safety

The first patient enrolled developed a skin reaction of rash and itchiness at the site of the wristband after 3 days of use. The patient stopped using the device, and the skin recovered without any treatment. Upon further evaluation, it was discovered that the patient had a pre-existing allergy to adhesives. The patient was withdrawn from the study by the investigator, and an exclusion criterion was added to the study to exclude patients with known allergy to adhesives. Subsequently, the electrode band was revised to minimize skin reaction. No other adverse events were reported in the study.

## Discussion

In this open-label pilot study, the TPNS system statistically significantly reduced tremor in patients with ET, and the only adverse effect was a minor skin reaction in one patient, suggesting that the AI-controlled TPNS may be a safe and effective treatment option for ET patients.

The currently approved TAPS device uses pre-patterned stimulation, with 2 daily sessions of stimulation recommended. In a pivotal study of 263 patients, 64% of patients reported residual tremor relief lasing an average of 94 minutes after the end of a 40-minute stimulation session [10]. In contrast, the TPNS system was worn throughout the daytime and there was no planning required to decide when the treatment would be administered. Instead, the AI algorithm in the TPNS system automatically made real-time adjustments to the stimulation parameters with the goal of providing full-day tremor relief, which was reflected in the reduction in TETRAS ADL scores. The findings from this study suggest that the more sustained delivery of stimulation with the TPNS system enables tremor reduction throughout the day.

The sample size was too limited for subgroup analysis. However, a few observations are worth noting. First, it was interesting to observe bilateral improvement in total TETRAS Performance Scale scores despite the unilateral stimulation in the majority (92%) of the patients, suggesting that the AI-driven stimulation in the TPNS system may lead to bilateral improvement from unilateral stimulation; however, this contralateral response will need to be confirmed in larger, controlled studies. Second, 3 patients with DBS participated in the study. One patient withdrew for personal reasons. For the 2 DBS patients who completed the follow-up, TETRAS PS dominant and nondominant hand scores improved by 2.5 (14.0 to 11.5) and 1.5 (12.8 to 11.3) points, respectively, while mADL improved by 3.5 (38.3 to 34.8) points, suggesting that the TPNS system may be an adjunctive treatment option for DBS patients.

The amount of missing data at the follow-up was higher than expected. However, other than the initial patient with a skin reaction that led to withdrawal from the study, no adverse effects were encountered. Other patients withdrew voluntarily due to personal reasons, such as having contracted COVID-19 or needing to care for a family member. In retrospect, the short follow-up of 7 to 10 days worked against study compliance because any temporary issue (such as illness) made it impossible for the patient to fulfill the study requirements, necessitating withdrawal.

Neither patients nor TETRAS raters were blinded, which may have led to subjective bias, and the lack of a sham control made it impossible to rule out any placebo effect. It is also possible that the device had a bracing effect that could have affected tremor, although we think this is unlikely given the light weight of the device (<75 g). Other limitations of this pilot study were the small sample size and short follow-up. Based on the results, the sponsor has performed a large, randomized, double-blinded (patient and rater), multiregion clinical trial comparing the TPNS system with a sham device, which will have up to 1 year of follow-up (NCT06235190).

## Conclusion

This is the first TPNS device that offers full-day, AI-driven stimulation for the treatment of ET. It was both safe and effective in this small pilot study, although the results from the ongoing large, randomized trial are needed for confirmation.

## Data Accessibility Statement

Data supporting this study cannot be made available because of the commercial interests of the study sponsor.
